# Hydrophobia

**Published:** 1858-10

**Authors:** 


### HYDROPHOBIA

FOLLOWS the bite of various animals; but more frequently that of a dog. There are two errors generally prevalent in reference to this most fearful of all diseases, which merit correction.

Hydrophobia is almost as frequent an occurrence outside of the "Dog-days," as during that period; and, second, mad dogs are not always afraid of the water, nor do they always exhibit a furious manner. The more certain signs of their being rabid, are: an unsteady walk, a haggard appearance, and an extraordinary and striking wildness in the expression of the eye. We, therefore, most earnestly advise, that whenever a person is bitten by any dog, even to the extent of the smallest scratch, whether in summer or winter, to saturate a rag instantly with common spirits of hartshorn, and sop it on the wound for at least half an hour, on the principle that all bites and stings owe their injurious effects to their acid nature, and hartshorn, being one of the strongest, simplest, and most accessible alkalies, is the most practicable antidote in Nature; the sooner it is applied, the more certain will be the success. The next most accessible thing of the same nature is, the liquor resulting from a cup of hot water poured on a handful of fresh ashes of wood.

